# Downregulation of TAB182 promotes cancer stem-like cell properties and therapeutic resistance in triple-negative breast cancer cells

**DOI:** 10.1186/s12885-023-11552-4

**Published:** 2023-11-13

**Authors:** Huan He, Shaozheng Wang, Wen Zhang, Shanshan Gao, Hua Guan, Pingkun Zhou

**Affiliations:** 1 Department of Radiation Toxicology and Oncology, Beijing Key Laboratory for Radiobiology, Beijing Institute of Radiation Medicine, Beijing, 100850 People’s Republic of China; 2 NHC Key Laboratory of Radiobiology, School of Public Health, Jilin University, Changchun, 130021 People’s Republic of China

**Keywords:** TAB182, Cancer stem cells, Cancer stemness property, Therapeutic resistance, Triple-negative breast cancer

## Abstract

TAB182 participates in DNA damage repair and radio-/chemosensitivity regulation in various tumors, but its role in tumorigenesis and therapeutic resistance in breast cancer remains unclear. In the current paper, we observed that triple-negative Breast Cancer (TNBC), a highly aggressive type of breast cancer, exhibits a lower expression of TAB182. TAB182 knockdown stimulates the proliferation, migration, and invasion of TNBC cells. Our study first obtained RNA-seq data to explore the cellular functions mediated by TAB182 at the genome level in TNBC cells. A transcriptome analysis and in vitro experiments enabled us to identify that TAB182 downregulation drives the enhanced properties of cancer stem-like cells (CSCs) in TNBC cells. Furthermore, TAB182 deletion contributes to the resistance of cells to olaparib or cisplatin, which can be rescued by silencing GLI2, a gene downstream of cancer stemness-related signaling pathways. Our results reveal a novel function of TAB182 as a potential negative regulator of cancer stem-like properties and drug sensitivity in TNBC cells, suggesting that TAB182 may be a tumor suppressor gene and is associated with increased therapeutic benefits for TNBC patients.

**Supplementary Information:**

The online version contains supplementary material available at 10.1186/s12885-023-11552-4.

## Introduction

Triple-negative breast cancer (TNBC), with low or negative ER, PR, or HER2 expression, is biologically heterogeneous, representing 10%-20% of all invasive breast cancers [1]. TNBC is more aggressive, has more advanced stages, and has higher rates of recurrence and metastasis than non-TNBC subtypes [1, 2]. Due to the lack of targeted agents, TNBC patient treatment is limited to cytotoxic chemotherapy [3]. However, patients with TNBCs are unlikely to achieve significant local- and disease-free survival advantages from adjuvant chemotherapy treatment in women [2, 4, 5]. The therapeutic strategy has recently changed with the advent of poly (ADP-ribose) polymerase (PARP) inhibitors (PARPis) for patients harboring BRCA mutations [1]. PARPis are thought to function by inhibiting DNA repair and replication in cancer cells deficient in BRCA1/2-dependent homologous recombination (HR) pathways through a process known as synthetic lethality [6]. Olaparib, a PARPi approved by the US Food and Drug Administration (FDA), selectively binds to and inhibits PARP. BRCA1-associated breast cancer is frequently a TNBC, but approximately 25% of TNBC patients carry a BRCA1 mutation, suggesting the limited application of PARPis [7]. Although olaparib monotherapy provides better median progression-free survival than single-agent chemotherapy, responses have not been highly durable, even in BRCA-mutant breast cancer patients [6, 8]. In addition, the development of drug resistance limits the efficacy of PARPis.

A similar situation has also been observed with among patients who received chemotherapy. Chemotherapy leads to an initial substantial response rate, followed by poor outcomes, such as frequent relapses and lower overall survival [9–11]. Cisplatin, which is a first generation platinum-based drug, is used to treat many solid tumors (e.g., lung, breast, and head and neck cancers) [12]. Platinum derivatives are alkylating agents that exert their effect by binding to DNA and inducing multiple single-strand breaks, resulting in apoptosis or other forms of cell death. Clinical trial data suggested that TNBC or other cancers patients with BRCA mutations exhibit more sensitivity to cisplatin and receive more benefits from cisplatin because of synthetic lethality [13, 14].

Therapeutic resistance is the main obstacle to TNBC patients experiencing satisfying outcomes. Moreover, the molecular mechanisms of therapeutic resistance are complex and interrelated among genomic and nongenomic factors [15–17]. Compared with ER- or HER2-positive breast cancer cells, TNBC cells display cancer stem-like cell (CSC) signatures at the functional, molecular, and transcriptional levels [18, 19]. TNBC aggressiveness has been associated, in part, with the breast cancer stem-like cells (ΒCSCs) that mediate tumor metastasis and contribute to the development of treatment resistance and recurrence. The reported molecular mechanisms underlying therapeutic resistance mediated by CSCs include the maintenance or acquisition of stemness and dormancy, increased DNA repair and drug efflux capacity, decreased apoptosis rates, and an interaction between CSCs and their supportive microenvironment, which is called the CSC niche [17, 20]. Therefore, therapies targeting CSCs are vital for achieving complete therapeutic responses and prolonging patient survival [19–21].

TAB182 was first identified as a novel 182 kDa tankyrase 1-binding protein by Seimiya H and colleagues in 2002 [22], and it was also named TNKS1BP1. TAB182 can directly bind to tankyrase 1 through its own ankyrin (ANK) domain and is identified by its RXXPDG motif [22, 23]. TAB182 is located in the nucleus and cytoplasm. Cytoplasmic TAB182 interacts with actin-capping proteins and is a negative regulator of cell motility and invasion [24]. In addition, TAB182 is a component of a larger mammalian CCR4-NOT protein complex, which can modulate helicase selective recruitment to the complex and shows the potential ability to determine the outcome of the targeted mRNA [25–27]. Additionally, several studies have reported that TAB182 participates in DNA double-strand break (DSB) repair and functions as a potential therapeutic target to increase the radio-/chemosensitivity of various tumors [28–31]. For instance, TAB182 modulates irradiation-induced DNA-PKcs phosphorylation and contributes to DNA DSB repair by regulating PARP-1/DNA-PKcs interaction [29]. However, the correlation between TAB182 and clinical outcomes is still unclear. The high expression level of TAB182 has been correlated with the poor survival outcomes of patients with lung cancer or esophageal squamous cell carcinoma (ESCC) [28, 30]. In the context of pancreatic cancer, TAB182 expression was lower in invasive regions than in normal and noninvasive regions [24]. TAB182 might be a prognostic marker and therapeutic target, but the precise roles of TAB182 in tumorigenesis have not been identified.

In this study, we selected MDA-MB-231 and BT549 cells, which are BRCA wild-type TNBC cells, to identify the functions of TAB182 in the development and progression of TNBC. We found that TAB182 expression was downregulated in TNBC cells, and TAB182 deletion increased the cell proliferation, colony formation, cell migration, and invasion, which suggested that TAB182 might act as a tumor suppressor gene in TNBC cells. Our paper first presents the gene expression profiles regulated by TAB182 in TNBC cells by RNA-seq assay. Both transcriptome analysis and in vitro experiments revealed that TAB182 plays a significant role in the development of cancer stemness in TNBC cells. Furthermore, TAB182 deletion contributes to the resistance of TNBC cells to olaparib and cisplatin by upregulating GLI2. GLI2 is a gene downstream in the Hippo signaling pathway, the most significant CSC-related pathway enriched by TAB182-regulated genes. Our results reveal a novel function of TAB182 as a prospective negative mediator of cancer stemness and resistance to olaparib or cisplatin in TNBC cells.

## Materials and methods

### Cell lines and treatments

Human breast cancer cell lines, including MCF7, ZR-75–1, MDA-MB-231, and BT549, and the human normal breast cell line MCF10A cell, were obtained from the American Type Culture Collection (ATCC). The MCF7, ZR-75–1, and MDA-MB-231 cells were cultured in DMEM (VCM5313, VIVICUMTM bioscience, Beijing, China) supplemented with 10% fetal bovine serum (FBS) and 1% penicillin–streptomycin (P/S). The BT549 cells were cultured in RPMI-1640 complete medium (PM150110B, Procell, Wuhan, China). The MCF10A cells were cultured in a medium specific for the MCF10A cell line (CM-0525, Procell, Wuhan, China). The cell lines were tested for mycoplasma. The cells were treated with olaparib (S1060, Selleck Chemicals) or cisplatin (S1166, Selleck Chemicals).

### shRNA, siRNA, and plasmids

TAB182 shRNA and negative control (NC) shRNA were cloned into the lentivirus vector LV3 (H1/GFP&Puro) (GenePharma, Suzhou, China). TNBC cells were infected with lentivirus harboring two TAB182 shRNAs, #1 and #2, or an NC shRNA, and after infection, the cells were selected after treatment with medium containing 2 μg/ml puromycin for approximately seven days, during which time, stable TAB182 knockdown MDA-MB-231 cells were generated.

TAB182-shRNA#1, sense: 5’-UAUCCAAGCGCUCUUCCCAAACUCC-3’, anti-sense: 5’-GGAGUUUGGGAAGAGCGCUUGGAUA-3’;

TAB182-shRNA#2, sense: 5’-AAGACGAGGAGUAAUCUUCACCCUG-3’, anti-sense: 5’-CAGGGUGAAGAUUACUCCUCGUCUU-3’.

The sequences of siRNA targeting TAB182 were as follows:

sense: 5’- GCCAAGACCAGAGUAAAGUTT-3’,

anti-sense: 5’-ACUUUACUCUGGUCUUGGCTT-3’.

The siRNA sequences targeting GLI2 were as follows:

sense: 5’-GCUUCACAUGACAGAUGUUTT-3’,

anti-sense: 5’-AACAUCUCUCAUCUGAAGCGG-3’.

For siRNA transfection, GP-transfect-Mate (G04009, GenePharma) was used.

To overexpress TAB182, cells were transfected with a pcDNA3.1^+^-TAB182-expressing vector constructed by GenePharma (Suzhou, China) using GP-transfect-Mate (G04009, GenePharma).

### Cell proliferation assay

CCK-8 (CK04, Dojindo Laboratories, Japan) was used to measure cell proliferation according to the manufacturer’s instructions. Cells with or without siRNA transfection for 72 h were seeded in a 96-well plate (1500 cells/well) and cultured for 0 days, 2 days, 3 days, and 4 days. To measure cell viability after olaparib or cisplatin treatment, 2000 cells/well were seeded in a 96-well plate, and after 24 h, they were treated with olaparib or cisplatin for 0 days, 1 day, 2 days, and 3 days. Cell proliferation was measured at the indicated time points by adding 10 µl/well CCK-8 reagent 3 h to the cultures before the measurement was taken. A Tecan Sunrise absorbance microplate reader was used to measure absorbance at 450 nm.

### Colony formation assay

A colony formation assay was performed to assess the cell clonogenic ability. Cells were plated into 6-well plates at 300 cells per well. After 24 h, the cells were treated with the indicated doses of olaparib (DMSO was the control) or cisplatin (ddH_2_O was the control). After culturing for 14 days, the cell clones were fixed with 4% paraformaldehyde for 15 min, stained with 0.1% crystal violet staining solution for 0.5 h -1 h, washed with PBS and dried. Images were acquired by scanning the plates using a scanner. The colonies (consisting of more than 50 cells) were counted by visual observation. The colony formation rate (%) was calculated according to the following formula: number of colonies per well/number of cells seeded per well × 100%.

### Invasion assay

For an invasion assay, Transwell chambers (8.0 μm pore size, Costar 3422, Corning incorporated) were precoated with Matrigel (#356,234, Corning) according to the manufacturer’s protocols. Cells were cultured in serum-starved medium overnight, and then 5 × 10^4^ cells/well were seeded in the upper chamber containing 200 µl serum-free DMEM. The lower chambers were filled with 600 µl DMEM complete medium. After 24 h of incubation at 37 °C, the cancer cells that penetrated the membrane were fixed with 4% paraformaldehyde for 15 min and stained with 0.1% crystal violet staining solution for 0.5 h -1 h. The chamber was washed with PBS solution, and the upper chambers were carefully cleaned with a cotton swab. After the chamber was dried, five random fields in each chamber were observed under a microscope (at 10 × magnification), and the number of invading cells per field was counted under a microscope.

### Wound healing assay

Cells were serum-starved for 24 h, trypsinized and seeded into 6-well plates at a density of 6 × 10^5^ cells/well in serum-free medium. After 24 h, a scratch wound was introduced in the across the cell monolayer with a sterilized 200 µl pipette tip. Cells migrating into the wounded area were observed at different time points (0 and 24 h) under an inverted light microscope at a magnification of × 10. In addition, five random fields in each well were observed under a microscope. Triplicate experiments were performed. ImageJ was used to measure the scratching wounds, and the migration rate was calculated according to the following formula: (areas at 0 h—areas at 24 h)/areas at 0 h × 100%.

### Sphere formation assay

Five thousand cells were resuspended in serum-free tumorsphere medium (CCM012, StemXVivo, R&D Systems) and then seeded as single cells into each well of a Nunclon Sphera 12-well plate (Thermo Scientific, 12–566-434) with a super low cell attachment surface. After 10–12 days of incubation in a 5% CO_2_ and 37 °C incubator, five random fields in each well were viewed under a microscope. The experiments were performed in triplicate. Then, the number of mammospheres (at 10 × magnification) greater than 20 μm diameter was counted using ImagJ software, and the quantification data are shown as the number of spheres per 5000 cells.

### Soft agar colony formation assay

A soft agar colony formation assay was performed according to a previously published protocol [32]. A total of 2000 cells were obtained as a single cell in the upper layer of agar of each well in a 6-well plate, and then, the plates were plated into a 37 °C humidified cell culture incubator. Two hundred microliters of culture medium was added to each well every three days to prevent desiccation. After approximately 21 days, images were taken under a microscope at 4 × or 10 × magnification. Colonies containing more than 50 cells were counted under a microscope at 4 × magnification, and the colony formation rate (%) was calculated according to the following formula: number of colonies per well/number of cells seeded per well × 100%.

### ALDEFLUOR assay

An ALDEFLUOR kit (#01700, StemCell Technologies, Canada) was used to measure ALDH enzyme activity. Diethylaminobenzaldehyde (DEAB), a specific inhibitor of ALDH, was used as the negative control. However, the TAB182 or control shRNAs used to generate stable TAB182 knockdown cells emitted GFP fluorescence, which overlapped with and interfered with the ALDH-positive fluorescence. Therefore, we used TAB182 siRNA-transfected, or TAB182-overexpressing TNBC cells to perform this assay. TNBC cells were transfected with 100 nM TAB182 siRNA or 2 μg pcDNA3.1^+^-TAB182-expressing plasmids in one well of a 6-well plate. After 72 h, the cells were processed for ALDEFLUOR assay according to the manufacturer’s protocols and then analyzed by NovoCyte 2060R Flow Cytometer.

### Western blotting

Total protein was extracted using M-PER™ Mammalian Protein Extraction Reagent (#78,501, Thermo Scientific). Equal amounts of protein (0.2 μg-1 μg) were loaded into the separation module kit (12–230 kDa or 66–440 kDa) and analyzed using an automated Simple Western system (Protein Simple WES) based on capillary electrophoresis technology to identify and quantify the levels of TAB182 (G-5, sc-514490, Santa Cruz, 1:50), GLI2 (C-10, sc-271786, Santa Cruz, 1:20), SOX2 (D6D9, #3579 s, CST, 1:20), and Slug (ab27568, Abcam, 1:20), according to the manufacturer’s instruction (Protein Simple, USA). Anti-α tubulin antibody (6A204, sc-69969, Santa Cruz, 1:100) or an anti-GAPDH antibody (TA-08, ZSGB-BIO, 1:100) was used as the internal control. Compass software (Protein Simple) was used to present the Western immunoblots.

### Quantitative RT‒PCR (qPCR)

Following the manufacturer's procedure, total RNA was extracted using the TRIzol reagent (Invitrogen, CA, USA). One microgram of RNA was reverse transcribed using the HiScript® III RT SuperMix for qPCR (+ gDNA wiper) (R323-01) (Vazyme Biotech, San Diego, USA). qPCR was performed on diluted cDNA with Taq Pro Universal SYBR qPCR Master Mix (Q712-02) (Vazyme Biotech, San Diego, USA). GAPDH was used as a reference gene, and the 2^−ΔΔCt^ formula was used to calculate relative expression. Primer sequences are as follows:

TAB182 forward: 5’- GGCCAGTAAAGTCTCCAGCA-3’;

TAB182 reverse: 5’- GTTGAAGGCCAGGTCGGAAG-3’.

GLI2 forward: 5’-GACATGCGACACCAGGAAGGAAGGT-3’;

GLI2 reverse: 5’-GCCGGATCAAGGAGATGTCAGAGATG-3’.

ALDH1A1 forward: 5’-CCAGGGCCGTACAATACCAA-3’;

ALDH1A1 reverse: 5’- CAGTGCAGGCCCTATCTTCC-3’.

GAPDH forward: 5’-GTCTCCTCTGACTTCAACAGCG-3’;

GAPDH reverse: 5’-ACCACCCTGTTGCTGTAGCCAA-3’.

### RNA-seq

RNA-seq experiments were performed in stable TAB182 knockdown cells or control cells. Experiments were performed in triplicate for each condition. LC Sciences (Hangzhou, China) conducted library construction and sequencing. Libraries were sequenced as t paired-end, 2 × 150 bp reads on an Illumina NovaSeq™ 6000 and aligned to the UCSC (http://genome.ucsc.edu/) Homo sapiens reference genome using the HISAT package. The mapped reads of each sample were assembled using StringTie. After the final transcriptome was generated, StringTie and EdgeR were used to estimate the expression levels of all transcripts. The data presented in this publication have been deposited in the NCBI Gene Expression Omnibus (GEO) database (GSE200038).

Both |fold change (FC)|> 1.5 and *P* values < 0.05 were considered to be the threshold to indicate differentially expressed genes for further analysis. Analysis of enrichments of genes in Gene Ontology (biologic processes, cell component, and molecular function) and KEGG pathway [33] were carried out with the online tool Database for Annotation, Visualization, and Integrated Discovery (DAVID, https://david.ncifcrf.gov/). *P* < 0.05 was considered significant.

### Statistical analysis

The data shown are the means with standard deviations (SD) from three independent experiments. An unpaired two-tailed Student’s t test was performed to compare the significance of the differences between the two groups. *P* < 0.05 was considered statistically significant.

The survival analysis was generated using the KM Plotter online tool (https://kmplot.com). The hazard ratio with 95% confidence intervals and the log-rank *P* value were calculated using Cox proportional hazards regression, and the two groups were separated on the basis of the median as the cutoff. Welch's test was performed to calculate the significance of a global difference between different groups for gene expression in different tissue subtypes using bc-GenExMiner v4.9 (http://bcgenex.ico.unicancer.fr/BC-GEM/). If a significant global difference was identified (*P* < 0.05) and there were more than two groups, Dunnett-Tukey–Kramer's test was computed for each pairwise comparison.

## Results

### ΤΑΒ182 deletion promotes cell growth, colony formation, cell migration, and invasion in TNBC cells

Knowledge about the functions of TAB182 is not comprehensive, especially the functions in tumors. ΤΑΒ182 has been reported to be associated with tumor aggression and metastasis. Nevertheless, inconsistent roles in different tumors or cells, such as pancreatic cancer, lung cancer, and esophageal squamous cell carcinoma, has been reported [24, 34, 35]. In this study, we aim to explore the roles of TAB182 in TNBC cells, a highly aggressive and metastatic subtype of breast cancer with poor survival outcomes.

**Fig. 1:**
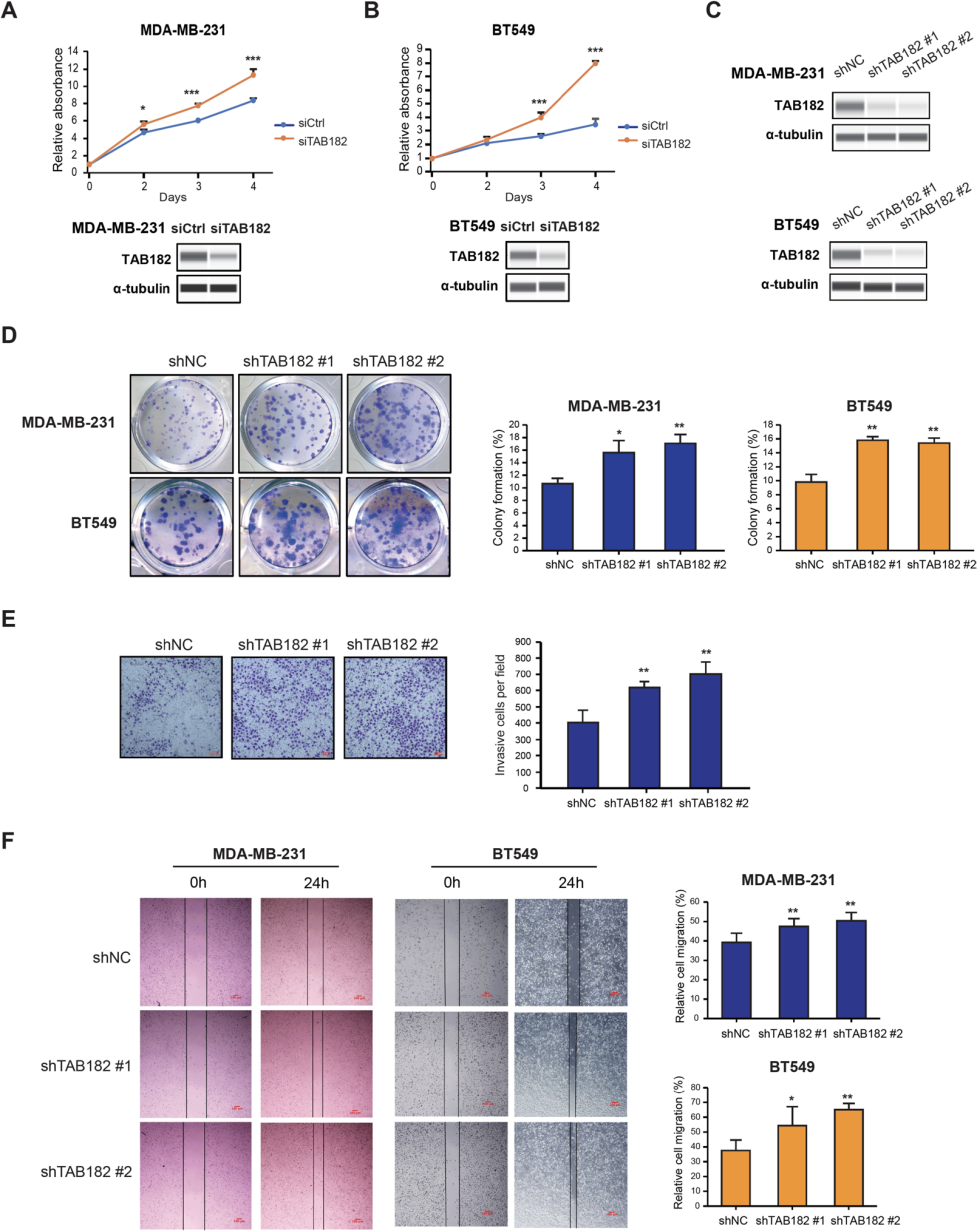
TAB182 deletion promotes cell growth, colony formation, migration, and invasion in TNBC cells. **A-B** CCK-8 cell proliferation assay was performed using TAB182 knockdown (siTAB182) or negative control (siCtrl) MDA-MB-231 cells (**A**) or BT549 cells (**B**) at the indicated time points. The knockdown of TAB182 was verified using Western blot analysis with the anti-TAB182 antibody. α-tubulin was used as the loading control. **C** Western blot analysis of TAB182 protein levels in stable TAB182 KD cells (shTAB182 #1 and shTAB182 #2) and control cells (shNC). α-tubulin was used as the loading control. **D** Cell colony formation assay revealed that TAB182 KD increased the colony formation ability compared with shNC cells, which was measured after seven days of incubation. The percentages of colony formation are presented in the right panel of D. **E** As determined with a Transwell assay, TAB182 depletion significantly promoted the invasion ability of MDA-MB-231 cells compared with shNC cells. Scale bar: 100 μm. Quantification data are presented as bar plots in the right panel of E. **F** The migratory ability of shTAB182 #1, shTAB182 #2, and shNC cells was measured by wound healing assay at 0 h and 24 h. Representative photos (10 × magnification, scale bar: 100 μm) and the relative cell migration percentages are presented as bar plots in the right panel of F. ^*^ *P* < 0.05, ^**^ *P* < 0.001 by Student’s t test. Error bars represent SD (*n* = 3)

By performing the CCK-8 cell proliferation assay in TNBC cells, we found that TAB182 knockdown (KD) promoted the proliferation of MDA-MB-231 and BT549 cells (Fig. 1A and B). The efficiency of siRNA-mediated TAB182 KD was confirmed by Western blot analysis (Fig. 1A and B, bottom). Then, we generated the stable TAB182 KD TNBC cell lines using two independent pairs of lentiviral vectors expressing shRNA against TAB182 (named shTAB182 #1 and shTAB182 #2) or negative control shRNA (shNC) in these two TNBC cell lines (Fig. 1C). Using stable TAB182 KD cells or control cells, we performed a cell colony formation assay and found that inhibition of TAB182 significantly increased the clonogenic ability of TNBC cells (Fig. 1D). Conversely, we overexpressed TAB182 in MDA-MB-231 cells transfected with the pcDNA3.1 + TAB182 plasmid (TAB182 O. E) or empty vector (Vec) (Fig. S1A, right panel), followed by CCK-8 assay and cell colony formation assay. The results verified the inhibitory role of TAB182 in regulating cell proliferation (Fig. S1A) and cell colony formation (Fig. S1B). Additionally, the results of a cell invasion assay indicated that the deletion of TAB182 markedly increased the cell invasion rate (Fig. 1E). Furthermore, the results of the wound healing assay showed that TAB182 KD increased the migration of in MDA-MB-231 and BT549 cells (Fig. 1F). Altogether, these results suggest that TAB182 can function as a negative regulator to reduce the proliferation, colony formation, cell invasion and migration of TNBC cells.

### TAB182 expression is downregulated in TNBC cells

**Fig. 2:**
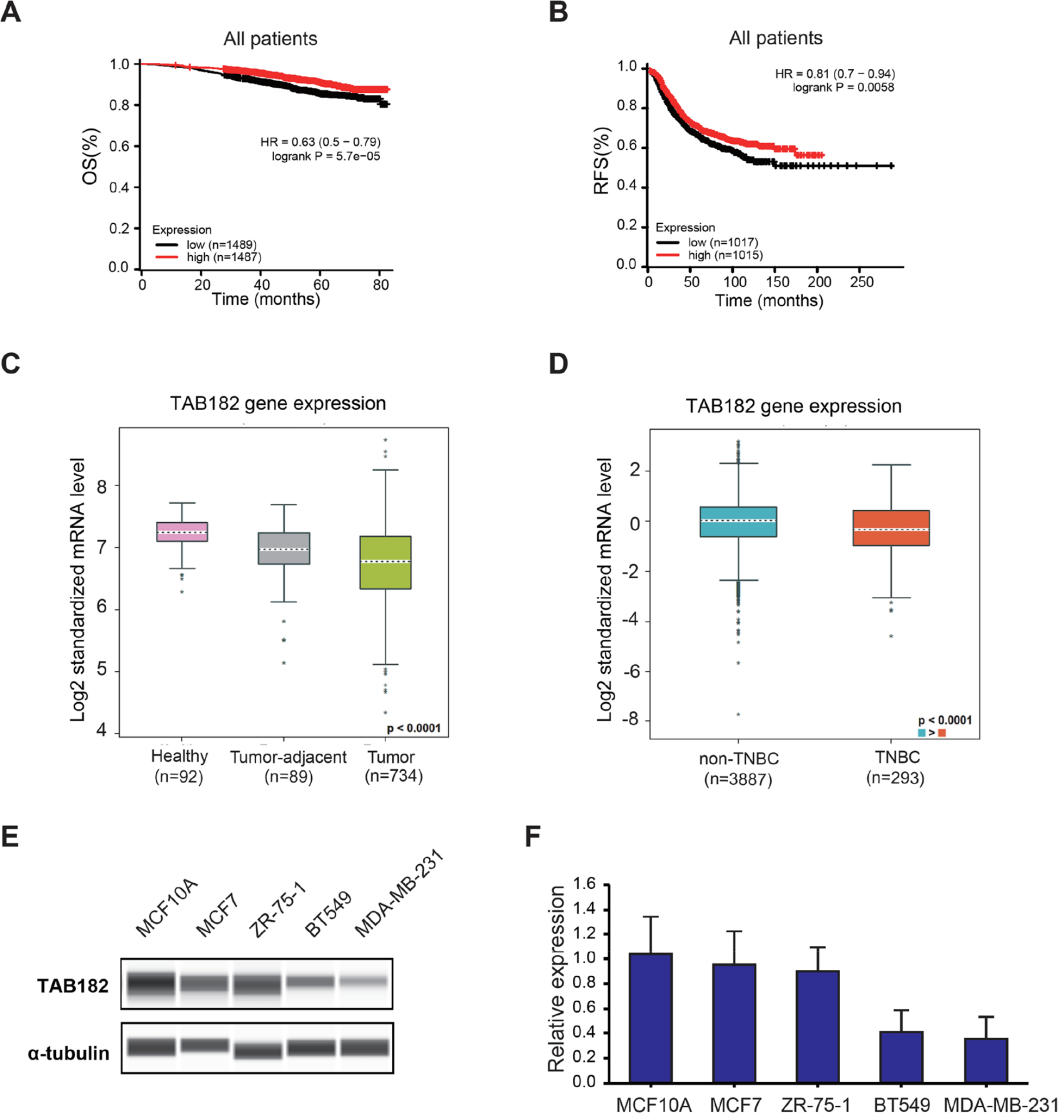
TAB182 expression is downregulated in TNBC cells. **A-B** Kaplan‒Meier plots showing the probability of overall survival (OS) (**A**) and relapse-free survival (RFS) (**B**) in all breast cancer patients, who were stratified on the basis of the median TAB182 (224792_at) gene expression level. **C-D** TAB182 mRNA levels among healthy, tumor-adjacent, and tumor samples (**C**). TNBC samples express lower mRNA levels of TAB182 than non-TNBC samples **D**. Analysis was performed using the TCGA RNA-seq databases from bc-GenExMiner v4.9. The *P* value shown in the figure indicates a significant global difference among three groups (**C**) or two groups (**D**). For each pairwise comparison (**C**), tumor-adjacent vs. healthy, *P* < 0.0001; tumor vs. healthy, *P* < 0.0001; tumor vs. tumor-adjacent, *P* < 0.01. **E** Western blot analysis results revealed TAB182 protein levels in a normal breast cell line (MCF10A), non-TNBC cell lines (MCF7 and ZR-751), and TNBC cell lines (BT549 and MDA-MB-231). **F** RT‒qPCR was used to determine TAB182 mRNA levels in the same cell lines shown in E. mRNA levels are relative to the mRNA expression of TAB182 in MCF10A cells. The data are shown as the means with SD (*n* = 3)

As limited studies on the role of TAB182 in breast cancer have been reported, we used the online public gene expression database to analyze the correlation between TAB182 expression and the survival outcomes of breast cancer patients. By analyzing the database (kmplot.com), elevated TAB182 gene expression was shown to be significantly associated with a higher possibility of overall survival (OS) (HR = 0.63, log-rank *P* value = 5.7e-05) or relapse-free survival (RFS) (HR = 0.81, log-rank *P* value = 0.0058) among breast cancer patients (Fig. 2A and B). Therefore, low expression levels of TAB182 may be associated with poor survival outcomes. Furthermore, using Breast Cancer Gene-Expression Miner v4.9 (http://bcgenex.ico.unicancer.fr/BC-GEM/), we observed that the mRNA expression level of TAB182 was lower in breast cancer samples than in healthy (*P* < 0.0001) or tumor-adjacent samples (*P* < 0.01) (Fig. 2C). Among breast cancer patients, TNBC patients had a lower expression level of TAB182 than non-TNBC patients (*P* < 0.0001) (Fig. 2D). We measured the protein or mRNA expression levels of TAB182 in various breast cell lines, including one human normal breast cell line (MCF10A) and breast cancer cell lines, TNBC cell lines (MDA-MB-231 and BT549), and non-TNBC cell lines (MCF7 and ZR-75–1). In addition, lower TAB182 expression was measured in TNBC cells, a finding that was confirmed, at both protein or mRNA levels compared with normal breast cells or non-TNBC cell lines (Fig. 2E and F). This finding indicates that the expression level of TAB182 is relatively low in highly aggressive and metastatic subtypes of breast cancer cells such as TNBC.

### Identification of gene expression profiles regulated by TAB182 in TNBC cells

**Fig. 3:**
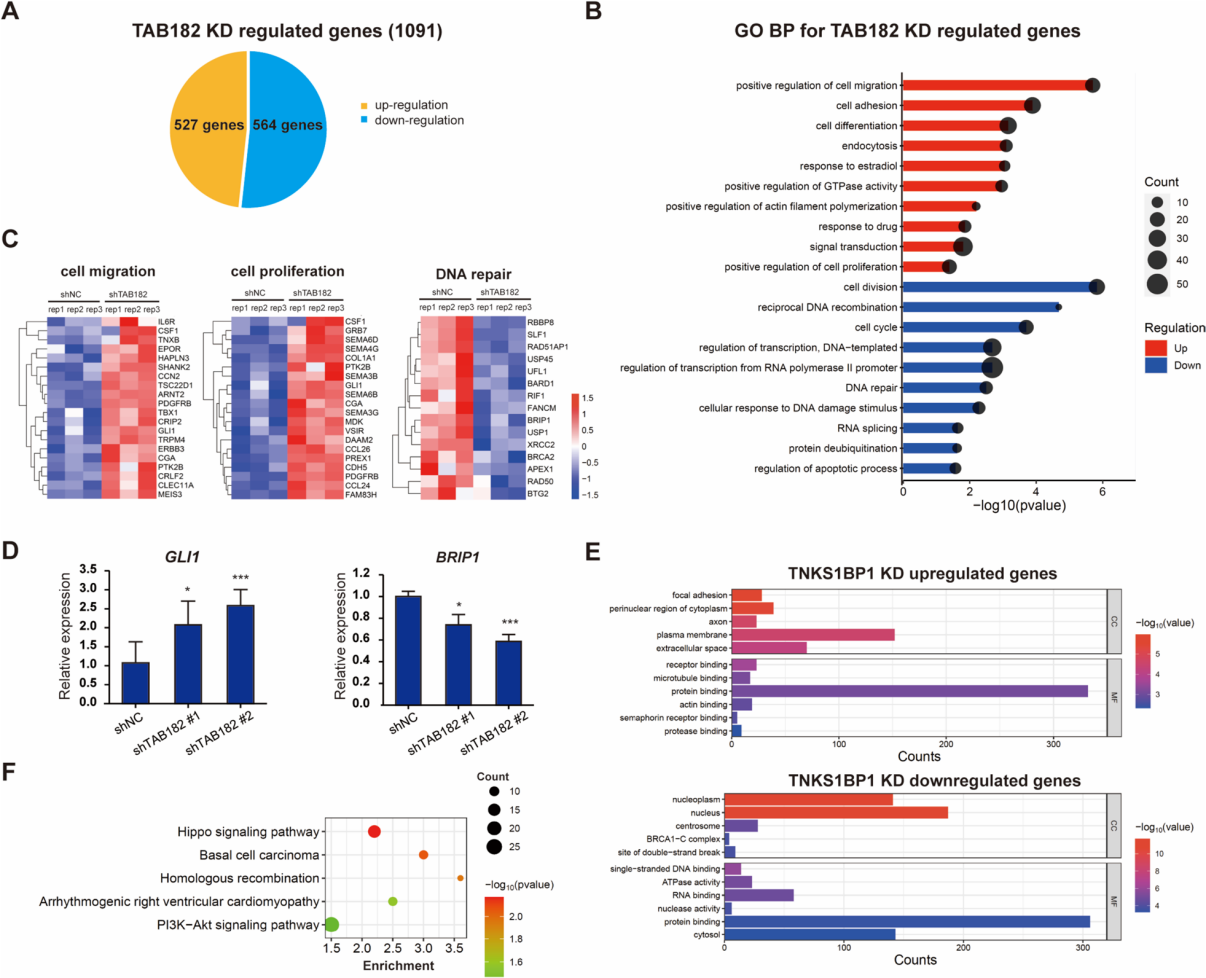
TAB182 regulated global gene expression in TNBC cells. **A** The pie chart presents the numbers of TAB182 KD-regulated genes (|Fold change|> 1.5, *P* < 0.05). **B** DAVID functional annotation shows the top 10 significant gene ontology biological processes (GO BP) for genes that were highly upregulated (upper) and downregulated (down) after TAB182 knockdown in MDA-MB-231 cells. *P* < 0.05. **C** Heatmaps showing the expression of TAB182 deletion-regulated genes enriched in three BP terms, including cell migration, cell proliferation, and DNA repair. The data are presented as transformed FPKM values of shTAB182 vs. shNC experiments. **D** A RT‒qPCR analysis confirmed the changes in gene expression identified by RNA-seq assay after TAB182 deletion. The data are shown as the means with SD relative to the control (shNC) from three independent experiments. ^*^ *P* < 0.05, ^***^ *P* < 0.001 by Student’s t test. **E** GO analysis of cell component (CC) and molecular function (MF) of the genes upregulated and downregulated after TAB182 deletion, respectively. **F** KEGG pathway analysis [33] revealed the signaling pathways significant enriched by TAB182 KD-regulated genes in MDA-MB-231 cells

To explore and characterize the functions of TAB182 in TNBC cells at the genome level, we performed RNA-seq analysis in one stable TAB182 KD and control MDA-MB-231 cell line. In a comparison of the transcriptome profiles, a total of 1091 genes were significantly regulated by TAB182 KD (|fold change|> 1.5, *P* < 0.05), among which 527 genes were upregulated and 564 genes were downregulated (Fig. 3A).

In agreement with the increased cell proliferation and cell migration observed in TAB182 KD cells, Gene Ontology (GO) analysis of TAB182 KD upregulated genes showed that genes related to cell proliferation and migration were highly enriched (Fig. 3B, upper). In addition, the genes downregulated by TAB182 KD were involved in DNA damage and repair-related biological processes, such as DNA repair, cell cycle, and cellular response to DNA damage (Fig. 3B, bottom), consistent with the known functions of TAB182. The expression levels of genes involved in cell migration, cell proliferation, and DNA repair are shown in the heatmaps in Fig. 3C. The influence of TAB182 KD on the mRNA levels of selected regulated genes (*GLI1* and *BRIP1*) was confirmed by quantitative RT‒PCR (Fig. 3D). *GLI1* contributes to both cell migration and cell proliferation processes, and *BRIP1* contributes to DNA repair (Fig. 3D). Regarding GO cellular components (CC) (Fig. 3E), the TAB182 KD upregulated genes were enriched in focal adhesion, perinuclear region of cytoplasm, axon, plasma membrane, and extracellular space. The changes in CC in the nucleoplasm, nucleus, centrosome, BRCA1-C complex, and site of double-strand break were observed in TAB182 KD downregulated genes. The GO molecular function (MF) terms indicated that TAB182 KD upregulated genes are closely related to receptor binding, microtubule binding, protein binding, etc. (Fig. 3E). The downregulated genes were closely associated with single-stranded DNA binding, ATPase activity, RNA binding, etc. (Fig. 3E). We then conducted a KEGG analysis (Fig. 3F), which revealed that the Hippo signaling pathway, basal cell carcinoma, homologous recombination, arrhythmogenic right ventricular cardiomyopathy, and PI3K-Akt signaling pathway were the predicted significantly enriched signaling pathways in TAB182 KD cells, consistent with the results of GO analysis.

### TAB182 overexpression increases olaparib or cisplatin sensitivity

It has been reported that TAB182 contributed to irradiation-induced DNA damage repair, and TAB182 upregulation was related to lung cancer cell resistance to irradiation or chemotherapy [28]. Our RNA-seq data indicated that the expression of the genes enriched in the DNA damage repair process was significantly inhibited by TAB182 deletion, which suggests that TAB182 KD may regulate the cell response to DNA-damaging anticancer drugs via synthetic lethality in TNBC cells. Although platinum compounds (e.g., cisplatin) or PARP inhibitors (e.g., olaparib) have been approved by the FDA for the clinical treatment of breast cancer, the benefit is limited to patients with defective DNA repair systems, such as those harboring BRCA mutations. Therefore, our study aimed to explore whether dysregulating TAB182 might expand the applications of cisplatin or olaparib by modifying drug sensitivity in BRCA-proficient TNBC cells (MDA-MB-231 and BT549).

**Fig. 4:**
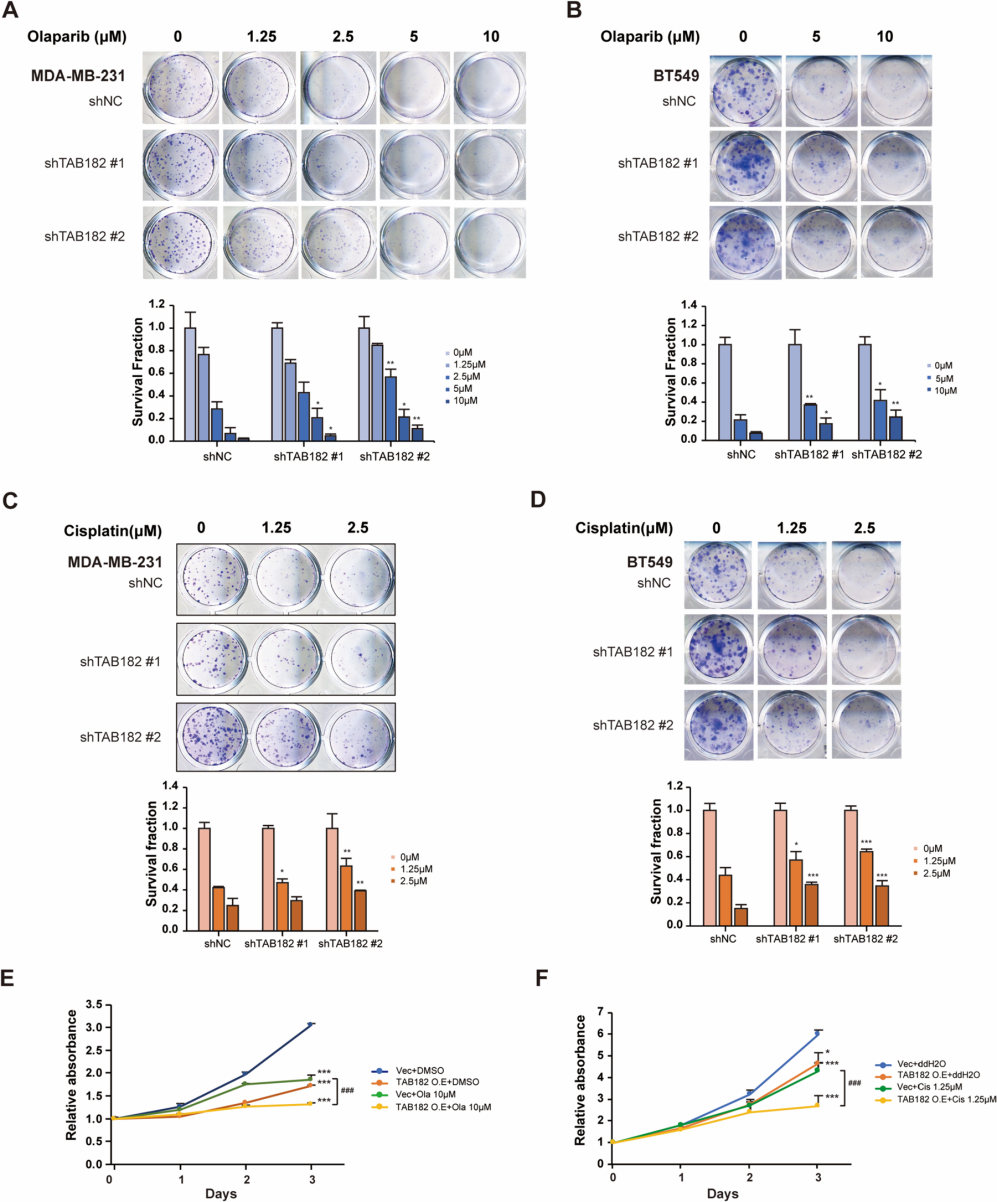
Downregulation of TAB182 causes cell resistance to olaparib or cisplatin treatment. **A-B** Colony formation assay in shTAB182 #1, shTAB182 #2, and shNC MDA-MB-231 cells (**A**) and BT549 cells (**B**) after olaparib or DMSO treatment for seven days at the indicated concentrations. ^*^ *P* < 0.05, ^**^ *P* < 0.01, shTAB182 #1 or #2 compared to the corresponding shNC after treatment with each concentration of olaparib, as determined by Student’s t test. The measurement of shNC-0 μM was used for normalization. The data are shown as the means with SD (*n* = 3). **C-D** Colony formation assay of shTAB182 #1, shTAB182 #2, and shNC MDA-MB-231 cells (**C**) and BT549 cells (**D**) after cisplatin or ddH2O treatment for seven days at the indicated concentrations. ^*^ *P* < 0.05, ^**^ P < 0.01 and ^***^ P < 0.001, shTAB182 #1 or #2 compared to the corresponding shNC after treatment with each concentration of cisplatin as determined by Student’s t test. The measurement of shNC-0 μM was used for normalization. The data are shown as the means with SD (*n* = 3). **E–F** CCK-8 assay using MDA-MB-231 cells overexpressing TAB182 (TAB182 O. E) or control cells (Vec) treated with 10 μM olaparib (Ola, **E**) or 1.25 μM cisplatin (Cis, **F**). DMSO or ddH2O was used as the control, and OD values were measured on Day 0, Day 1, Day 2, and Day 3 after treatment. The absorbance measured on Day 0 was used for normalization. The data are shown as the means with SD (*n* = 3). ^*^ *P* < 0.05, ^***^ *P* < 0.001, compared with Vec + DMSO/ddH_2_O; ^###^ *P* < 0.001, TAB182 O. E + Ola/Cis vs. Vec + Ola/Cis, by Student’s t test (*n* = 3)

First, we performed a colony formation assay and found that TAB182 KD MDA-MB-231 cells exhibited resistance to olaparib (Fig. 4A). After 5 μM and 10 μM olaparib treatment, TAB182 deletion significantly increased the cell survival fraction of both shTAB182 #1 and shTAB182 #2 cells compared with that of shNC cells (Fig. 4A). Similarly, the downregulation of TAB182 inhibited the sensitivity of BT549 cells to olaparib compared to that of shNC cells (Fig. 4B). The same effect of TAB182 was observed in both TNBC cell lines after cisplatin treatment (1.25 μM and 2.5 μM) (Fig. 4C and D). Additionally, we overexpressed TAB182 in MDA-MB-231 cells and examined cell viability 0, 1, 2 and 3 days after olaparib (10 μM) or cisplatin (1.25 μM) treatment using a CCK-8 assay (Fig. 4E and F). When olaparib treatment was combined with TAB182 overexpression, the cell growth rate was significantly reduced compared with that of Vec cells (Fig. 4E). Similarly, TAB182 overexpression enhanced the inhibitory effect of cisplatin on cell survival (Fig. 4F). Thus, the downregulation of TAB182 is associated with cell resistance to DNA-damaging agents in TNBC cells.

### TAB182 deletion contributes to the characteristics of BCSCs

According to the aforementioned results, we found that the downregulation of TAB182 enhanced cell proliferation, cell invasion, cell migration, and chemoresistance, which were associated with CSCs. Moreover, analysis of the RNA-seq data indicated that the most significant pathway enriched by TAB182 KD-regulated genes was the Hippo signaling pathway (Fig. 3F). This pathway has been reported to be associated with CSCs, and CSCs are the main contributor to the cancer aggressive process and drug resistance.

**Fig. 5:**
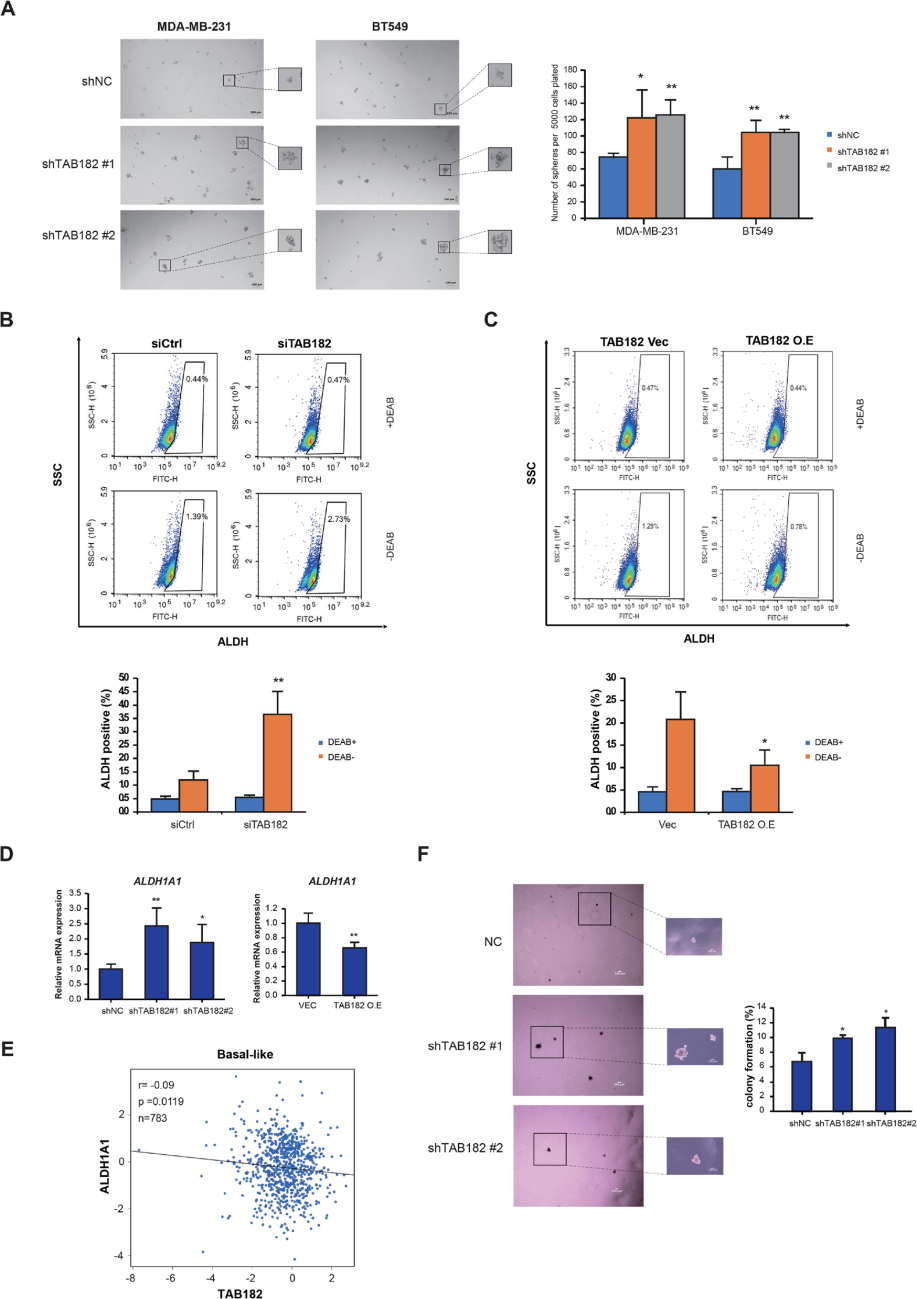
TAB182 deletion contributes to the acquisition of breast cancer stem-like cell properties. **A** Sphere formation assays were performed with stable TAB182 KD (shTAB182 #1 and shTAB182 #2) and shNC TNBC cells. Representative photos (10 × magnification, scale bar: 100 μm) and quantification data are presented. ^*^ *P* < 0.05, ^**^ *P* < 0.01 by Student’s t test. Error bars represent SD (*n* = 3). **B-C** An ALDEFLUOR assay was performed to measure changes in the percentages of the ALDH-positive population in TNBC cells after TAB182 was deleted (**B**) or overexpressed (**C**). DEAB was used as the negative control. SSC: side scatter. FITH: the fluorescence signal of ALDH. The percentages of ALDH-positive cells are presented at the bottom of B and C. ^*^ *P* < 0.05, ^**^ *P* < 0.01, DEAB-siTAB182/TAB182 O.E vs. DEAB-siCtrl/Vec, by Student’s t test. **D** RT‒qPCR was used to determine ALDH1A1 mRNA levels in TAB182 KD or TAB182 O.E cells. mRNA levels are relative to the mRNA expression level of TAB182 in shNC or Vec cells. The data are shown as the means with SD (n = 3). ^*^ *P* < 0.05, ^***^ *P* < 0.001 by Student’s t test. **E** Scatter plot showing significant Pearson’s correlation between TAB182 and ALDH1A1 mRNA levels in basal-like breast cancer (*n* = 783) with data obtained from the online database bc-GenExMiner v4.9. **F** TAB182 knockdown increased the colony formation rate of MDA-MB-231 cells in soft agar (4 × and 10 × magnification, scale bar: 100 μm), with quantification results presented in a bar plot. ^*^ *P* < 0.05 compared with the shNC group by Student’s t test (*n* = 5)

Therefore, we performed a mammosphere formation assay to assess the role of TAB182 in the biological processes of BCSCs. The TAB182 KD MDA-MB-231 or BT549 cells exhibited an increased number and size of mammospheres compared with those of shNC cells, indicating that TAB182 downregulation resulted in increased tumorigenesis potential (Fig. 5A). Fluorescence-activated cell sorting (FACS) was performed to sort cells expressing high levels of ALDH, which represent the BCSC population. TAB182 KD increased the percentage of ALDH-positive MDA-MB-231 (2.73% vs*.* 1.39%) (Fig. 5B) and BT549 cells (2.07% vs*.* 1.36%) (Fig. S2A). In contrast, TAB182 overexpression resulted in a significantly reduced percentage of ALDH-positive cells (Fig. 5C and Fig. S2B). Additionally, we examined changes in ALDH1A1 expression, a key ALDH isotype linked to CSCs, after TAB182-induced dysregulation. Compared with that in control cells, the expression of ALDH1A1 mRNA was elevated in TAB182-deleted cells and was downregulated in TAB182-overexpressed cells (Fig. 5D). Using an online RNA-seq database based on basal-like breast cancer patients, we found that TAB182 negatively correlated with ALDH1A1 at the gene expression level in basal-like breast cancer patients (Fig. 5E). TAB182 KD increased the expression levels of cancer stem cell-related proteins, SOX2 and Slug (Fig. S3A), using Simple Western assay. Reversely, overexpressing TAB182 can inhibit their expression (Fig. S3B). Moreover, by performing a soft agar assay, we found that the deletion of TAB182 increased the size of cell colonies and the percentage of cells that formed colonies, indicating that low expression of TAB182 promoted cell self-renewal, a property characteristic of CSCs (Fig. 5F).

### TAB182 deletion results in drug resistance by increasing GLI2 expression

**Fig. 6:**
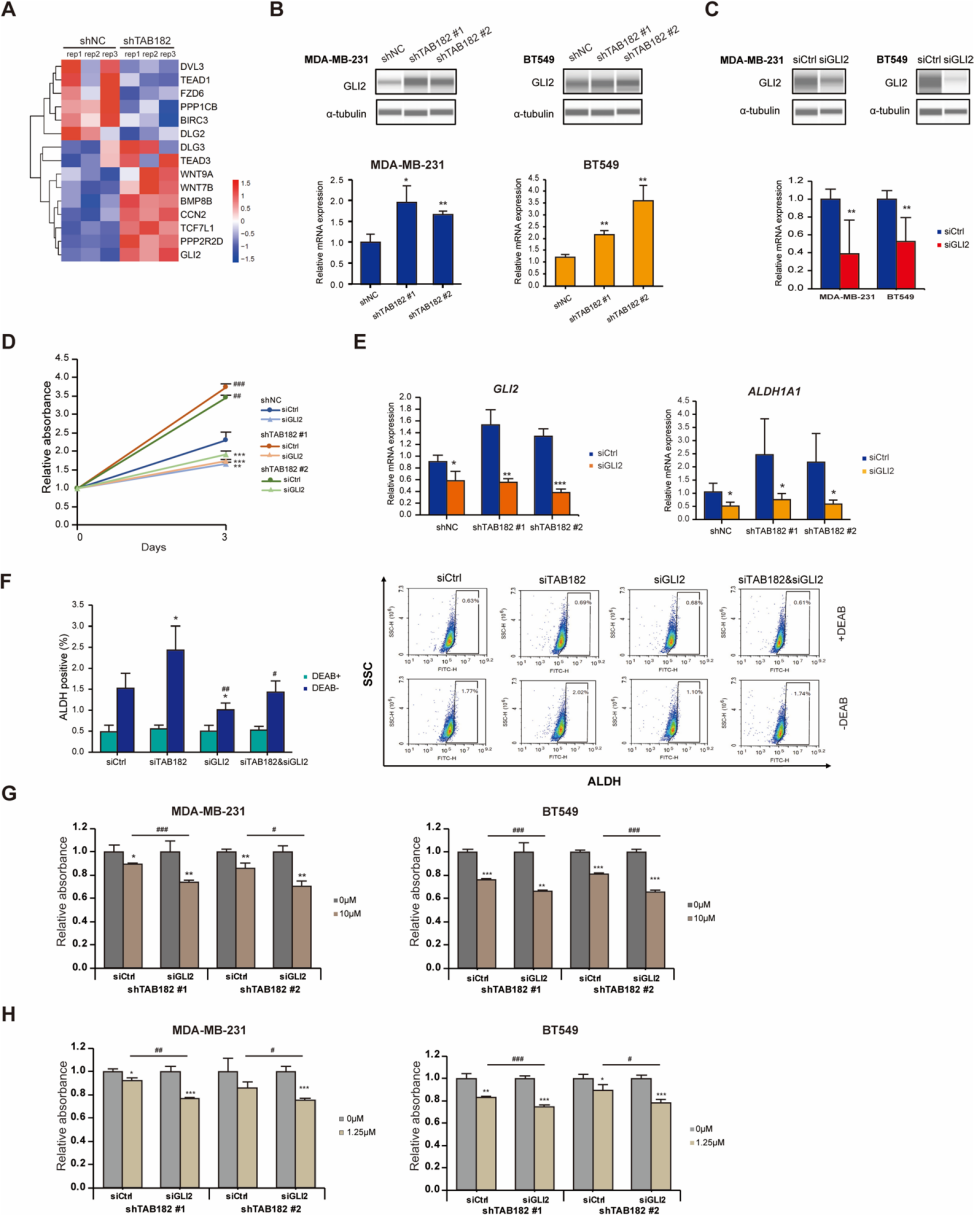
TAB182 deletion drives olaparib or cisplatin resistance by upregulating GLI2. **A** Heatmaps show the expression of a TAB182 deletion-regulated gene set enriched in the Hippo signaling pathway. The data are presented as transformed FPKM values of shTAB182 vs. shNC experiments.** B** The protein and mRNA levels of GLI2 upregulated by TAB182 knockdown were measured by Western blot analysis (top) and RT‒qPCR (bottom), respectively. α-tubulin was used as the loading control. The data are shown as the means with SD (*n* = 3). ^*^ *P* < 0.05, ^**^ *P* < 0.01 by Student’s t test. **C** Knockdown of GLI2 significantly reduced GLI2 expression at both the protein (top) and mRNA levels (bottom) 72 h after siRNA transfection in MDA-MB-231 and BT549 TNBC cells. α-tubulin was used as the loading control. The data are shown as the means with SD (*n* = 3). ^**^ *P* < 0.05 by Student’s t test. **D** CCK-8 assay using shTAB182 #1, #2, or NC cells, which were transfected with GLI2 (siGLI2) or control siRNA (siCtrl). After three days, OD was measured, and the absorbance values were compared relative to those obtained on Day 0. **E** RT‒qPCR analysis reveals that GLI2 or ALDH1A1 gene expression changes after GLI2 deletion in shTAB182 #1, #2, or NC cells. The data are shown as the means with SD relative to control (shNC cells transfected with siCtrl) from three independent experiments. ^*^ *P* < 0.05, ^***^ *P* < 0.001, siGLI2 vs. siCtrl, by Student’s t test. **F** The proportion of ALDH-positive cells was determined using an ALDEFLUOR assay kit and GLI2-deleted shNC, shTAB182 #1, and shTAB182 BT549 cells, and it was followed by a flow cytometry analysis. DEAB was used as the negative control. SSC: side scatter. FITH: the fluorescence signal of ALDH. The percentages of ALDH-positive cells are presented in the left panel of F. ^*^ *P* < 0.05, ^**^ *P* < 0.01, compared with siCtrl/Vec by Student’s t test. **G-H** siGLI2- or siCtrl-transfected stable TAB182 KD cells (shTAB182 #1 and shTAB182 #2) were treated with olaparib (Ola, 10 μM) (**G**) or cisplatin (Cis, 1.25 μM) (**H**). OD was measured 72 h after treatment, and the absorbance value obtained after DMSO treatment was used for normalization. The data are shown as the means with SD (*n* = 3). ^*^ *P* < 0.05, ^**^ *P* < 0.01, ^***^ *P* < 0.001, compared with DMSO; ^#^ *P* < 0.05, ^##^ *P* < 0.01, ^###^ *P* < 0.001, siGLI2 with Ola/Cis treatment vs. siCtrl with Ola/Cis treatment, by Student’s t test

Among TAB182 KD upregulated genes enriched in the Hippo signaling pathway, we selected *GLI2* (log_2_FC = 0.95, *P* < 0.001 in RNA-seq data) (Fig. 6A), a downstream gene of the Hippo signaling pathway that plays an essential role in modifying the properties of CSCs. The expression of GLI2 was validated by Western blot analysis and RT‒qPCR in MDA-MB-231 and BT549 cells (Fig. 6B). To analyze how GLI2 affects BCSCs and drug sensitivity in TNBC cells, we knocked down GLI2 using siRNA and validated the deletion of GLI2 by RT‒qPCR and Western blot analysis (Fig. 6C). As shown in the results of the CCK-8 assay presented in Fig. 6D, deleting GLI2 significantly suppressed the stimulating effect of TAB182 deletion on cell proliferation. In addition, when the mRNA levels of *GLI2* were reduced by siRNA in TAB182 KD TNBC cells, the elevated expression of *ALDH1A1* was attenuated (Fig. 6E). After deleting GLI2, the increased proportion of ALDH-positive cells was reduced in TAB182 KD cells (Fig. 6F), which suggested that TAB182 modifies the characteristics of BCSCs by regulating GLI2 expression. Therefore, we examined cell viability after olaparib or cisplatin treatment in GLI2 siRNA-transfected TAB182 KD cells to determine whether GLI2 KD reverses the effects of TAB182 KD on drug resistance. After olaparib stimulation, siGLI2 counteracted the effect of TAB182 deletion and significantly reduced the viability of the stable TAB182 KD cells compared with control cells (siCtrl) (Fig. 6G). Similar results were obtained after cisplatin treatment (Fig. 6H). GLI2 inhibition increased the inhibitory effect of cisplatin on TAB182 KD cell proliferation. Together, these results indicate that the downregulation of TAB182 results in the development of BCSCs and drug resistance in TNBC cells by increasing GLI2 expression.

## Discussion

To date, the published literature on the roles of TAB182 has been focused mainly on radioresistance driven by TAB182 contributions to DNA repair in various tumors. There is no comprehensive understanding of the functions of TAB182 in tumorigenesis, especially in breast cancer. Although TAB182 has been reported to be associated with tumor aggression and metastasis, reports of its roles in different tumors or cells have been inconsistent [24, 33]. In this study, we found that downregulation of TAB182 increases the proliferation, colony formation, migration, and invasion of TNBC cells, consistent with the study of T. Ohishi and colleagues [24], who found a low expression level of TAB182 in pancreatic cancer cells and found that TAB182 deletion played an essential role in cell motility and invasion. However, Gao A et al. found that the downregulation of TAB182 inhibited ESCC cell invasion and migration, and TAB182 was expressed at a high level in ESCC compared to normal cells or tissues [33]. These contradictory findings suggest that TAB182 may play distinct roles in different types of cancer cells and may be associated with the different basal expression levels of TAB182 in cancer cells compared to that in normal cells or tissues. Therefore, we analyzed the mRNA expression level of TAB182 using online databases. Compared to normal breast tissues or non-TNBC samples, a lower expression level of TAB182 was found in TNBC. Then, we confirmed the downregulation of TAB182 in TNBC cell lines at the protein and mRNA level. Cell migration and invasion are required for cancer cell metastasis, and cancer metastasis is the main reason for mortality in breast cancer patients. These findings suggested that TAB182 may function as a tumor suppressor gene in TNBC cells by inhibiting cell proliferation, colony formation, and cell invasion and migration.

Since the aforementioned results show clear cell phenotypes acquired after TAB182 KD in TNBC cells, the downstream signaling associated with TAB182 deletion was evaluated at the transcriptome level. Our study confirmed the function of TAB182 at the genome level via RNA-seq analysis, which provided information the gene expression profiles modified by TAB182, enabling further study of its regulatory mechanisms. The functional enrichment analysis results revealed that genes upregulated by TAB182 deletion were enriched in cell proliferation and positive regulation of cell migration biological processes, which strongly supports the acquisition of a functional phenotype of TAB182 in TNBC cells. In addition, we found that TAB182 depletion upregulated genes significantly associated with the positive regulation of actin filament polymerization, such as *CCL24* and *CCL26*, which play essential roles in cancer cell invasion and migration [36, 37]. Furthermore, the expression of *ICAM1* or *TGFBI* has been shown to be upregulated in TNBC and related to tumor aggressiveness and metastasis [38–40], and the expression of both genes were increased in TAB182 KD cells and participated in the cell adhesion process in the present study. The knockdown of TAB182 inhibited the expression of genes that participate meaningfully in the DNA damage and repair signaling pathways, consistent with the known function of TAB182 in DNA damage repair after ionizing radiation (IR) or adenovirus infection [28, 31]. The results of the KEGG analysis showed that TAB182-regulated genes participated in the homologous recombination signaling pathway, which is one of the critical pathways involved in the repair of double-strand DNA damage. Moreover, the set of genes downregulated by TAB182 deletion was associated with cell cycle process, which corresponds to a recent study indicating that TAB182 downregulation hindered IR-induced G2/M arrest [30]. Additionally, our RNA-seq data provide more information to further explore unknown or novel functions of TAB182. For instance, the dysregulation of TAB182 modified the Hippo signaling pathway and the PI3K-Akt signaling pathway, which play critical roles in regulating various cellular functions, including cell growth and proliferation, and their dysregulation has been implicated in several diseases, including cancer [41, 42].

For TNBC patients, chemotherapy (e.g., cisplatin) has been the main treatment option for a long time. Recently, the therapeutic strategy has changed with the advent of PARP inhibitors (e.g., olaparib) for patients harboring a mutation in the BRCA genes [7, 13]. Although chemotherapy or PARP inhibitors lead to an initial substantial response, most patients inevitably develop resistance [9–11]. Therapeutic resistance is a significant barrier to complete breast cancer management and is often followed by poor outcomes, such as frequent relapses and lower overall survival [43]. Dysregulation of the DNA damage repair process is vital to drug sensitivity, including sensitivity to genotoxic agents or DNA-damaging anticancer drugs [44]. Therefore, we explored the effects of TAB182 on drug sensitivity in the MDA-MB-231 and BT549 cell lines, which are BRCA-wild-type TNBC cell lines, and compared the results to those obtained with BRCA-mutated cells, which exhibit intrinsic resistance to olaparib or cisplatin [45, 46]. Our study indicates that the overexpression of TAB182 increased the inhibitory effects of olaparib or cisplatin on cell viability more than either treatment alone, which indicates that TAB182 expression negatively regulates therapeutic resistance in TNBC cells. This is inconsistent with the roles of TAB182 in A549 lung cancer cells [28]. How TAB182 downregulation results in drug resistance in TNBC cells remains to be investigated.

An integrative analysis of the TNBC cell phenotypes and gene expression profiles induced by TAB182 deletion suggested that TAB182 might regulate the formation of CSCs. The downregulation of TAB182 increased cell proliferation, cell invasion, and cell migration and chemoresistance, which are functional outcomes induced by the presence of CSCs. In this study, a KEGG pathway analysis indicated that the expression of gene sets regulated by TAB182 deletion was significantly enriched in cancer stemness-related pathways, namely, the Hippo signaling pathway and the PI3K-AKT signaling pathway. Both of these pathways play vital roles in regulating stemness in various cancers, including TNBC [15, 47]. According to the literature, the CSCs represent the major sources of malignant progression and poor prognosis of tumors because of their features which differ from those non-CSCs, such as activation or acquisition of self-renewal ability and establishment of a heterogeneous population of tumor cells after treatment [48]. In our study, the properties of CSCs were examined using in vitro functional experiments. Sphere formation and soft agar colony formation assays demonstrated that TAB182 deregulation increased the tumorigenic and self-renewal abilities of TNBC cells. Furthermore, the percentage of CSCs decreased after TAB182 overexpression. Our study showed that TAB182 plays a considerable role in developing the cancer stem-like properties of TNBC cells.

Various studies have reported that dysregulation of some breast stem cell markers (e.g., ALDH1A1) or signaling pathways (e.g., Hippo/YAP pathway or Hedgehog signaling) relieves drug resistance in different cancers [17, 49–51]. Here, we assumed that TAB182 deletion induced therapy resistance related to cancer stemness. Based on our RNA-seq data, we focused on the Hippo signaling pathway, the most significant pathway enriched by the TAB182-KD-regulated gene set. Among the genes in this gene set, GLI2 was markedly upregulated by TAB182-KD (fold change = 1.93, *P* < 0.001), which was verified by Western blot and RT‒qPCR analysis in this study. GLI2 is a downstream target gene of the Hippo/YAP signaling pathway and a transcriptional activator of Hedgehog signaling, and the two aforementioned pathways are essential regulators of CSC maintenance [52, 53]. GLI2 has been reported to affect stemness and drive chemoresistance in various cancers [54–59]. For instance, in colorectal cancer, the hypoxic tumor microenvironment activates the expression of GLI2 in CSCs, resulting in increased stemness/dedifferentiation and intrinsic resistance to chemotherapy [54]. In our study, we inhibited GLI2 expression by siRNA, which reversed the increase in the cell proliferation rate that had been induced by TAB182 deletion. In addition, GLI2 deletion inhibited the expression level of ALDH1A1 and the percentage of ALDH-positive cells independent of TAB182 KD. These findings suggest that TAB182 KD promotes CSC development by stimulating GLI2 expression. After olaparib or cisplatin treatment, inhibition of GLI2 overcame cell resistance induced by lower expression of TAB182. Taken together, these results indicate that low expression of TAB182-induced tumorigenesis and therapeutic resistance might be mediated through cancer stemness signaling pathways, and GLI2 shows the potential to be a target and leveraged to reduce cancer stemness in TAB182 low-expression breast cancers.

The limitation of our study is that we explored only the functions of TAB182 via in vitro experiments, and the results need to be validated through in vivo experiments. Additionally, the regulatory effects of TAB182 KD on GLI2 expression need to be further investigated to confirm the specific mechanisms. There is still an urgent need for extensive research on therapy resistance to develop novel biomarkers and therapeutic targets that can predict therapeutic responses or improve the clinical outcomes of TNBC patients. However, the molecular mechanisms of therapeutic resistance are complex and interrelated, involving both genomic and nongenomic factors [15–17]. For the clinical application of TAB182 to be realized, we aim to identify the core mechanisms involved in regulating TAB182 deletion-driven cell stemness and therapy resistance in our further study.

In summary, our results reveal gene expression profiles regulated by TAB182 and identify TAB182 has a possibility to act as a novel negative regulator related to the development of cancer stem-like properties and olaparib/cisplatin resistance that regulates GLI2 expression in the BRCA-proficient TNBC cell lines. Our findings suggest that TAB182 may be a tumor suppressor gene and a potential therapeutic target for TNBC patients.

### Supplementary Information

**Additional file 1: Figure S1. **Overexpression of TAB182 inhibits cell proliferation and colony formation. **Figure S2.** TAB182 deletion increases the percentage of ALDH-positive cells. **Figure S3.** Deleting TAB182 enhances the expression of cancer stemness-related protein markers.

## Data Availability

The RNA-seq datasets generated and/or analysed during the current study have been deposited in the GEO repository, [GSE200038].
