# Supplementary material for: Downregulation of TAB182 promotes cancer stem-like cell properties and therapeutic resistance in triple-negative breast cancer cells
Source: BMC Cancer. 2023 Nov 13;23:1101. doi: 10.1186/s12885-023-11552-4 (PMC10642046; doi:10.1186/s12885-023-11552-4)
Supplement: Supplementary file 1 — Additional file 1: Figure S1. Overexpression of TAB182 inhibits cell proliferation and colony formation. Figure S2. TAB182 deletion increases the percentage of ALDH-positive cells. Figure S3. Deleting TAB182 enhances the expression of cancer stemness-related protein markers. [file 12885_2023_11552_MOESM1_ESM.docx]

**Supplementary Materials**

Figure S1. Overexpression of TAB182 inhibits cell proliferation and colony formation.

Figure S2. TAB182 deletion increases the percentage of ALDH-positive cells.

Figure S3. Deleting TAB182 enhances the expression of cancer stemness-related protein markers.

**
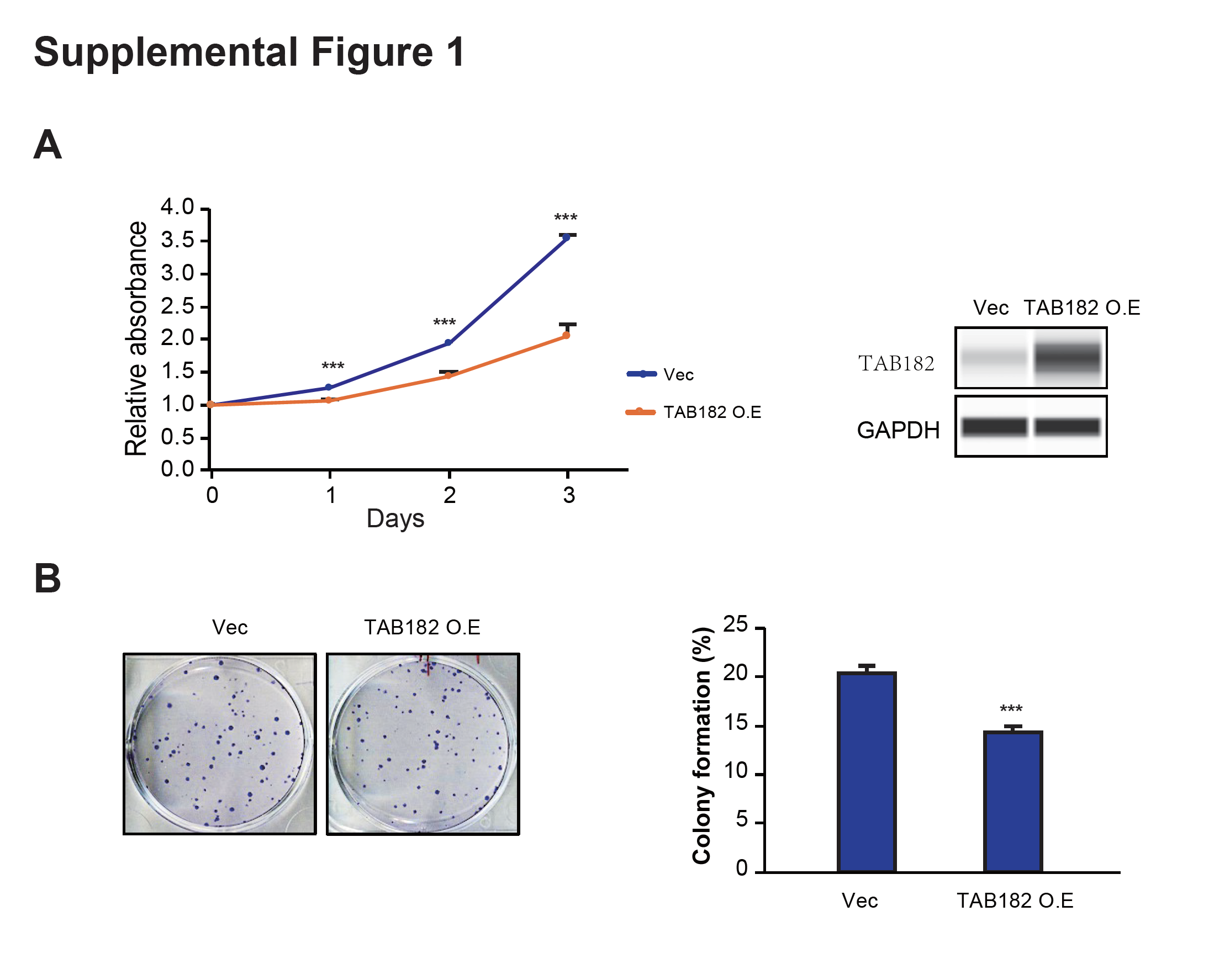
**

**Fig. S1. Overexpression of TAB182 inhibits cell proliferation and colony formation. (A)** CCK-8 cell proliferation assay was measured at indicated time points in MDA-MB-231 cells transfected with pcDNA3.1+ TAB182 overexpression plasmid (TAB182 O.E) or Empty vector. Absorbance measured at day 0 was used for normalization. ^***^ *P*<0.001 by Student’s t test (n=3). The overexpression of TAB182 was verified by Western blot analysis using the anti-TAB182 antibody, and GAPDH was used as the loading control (right panel of **C**). **(B)** The representative photos and quantification results of cell colony formation assay in TAB182-overexpressed MDA-MB-231 cells. ^***^ *P*<0.001 by Student’s t test (n=3).


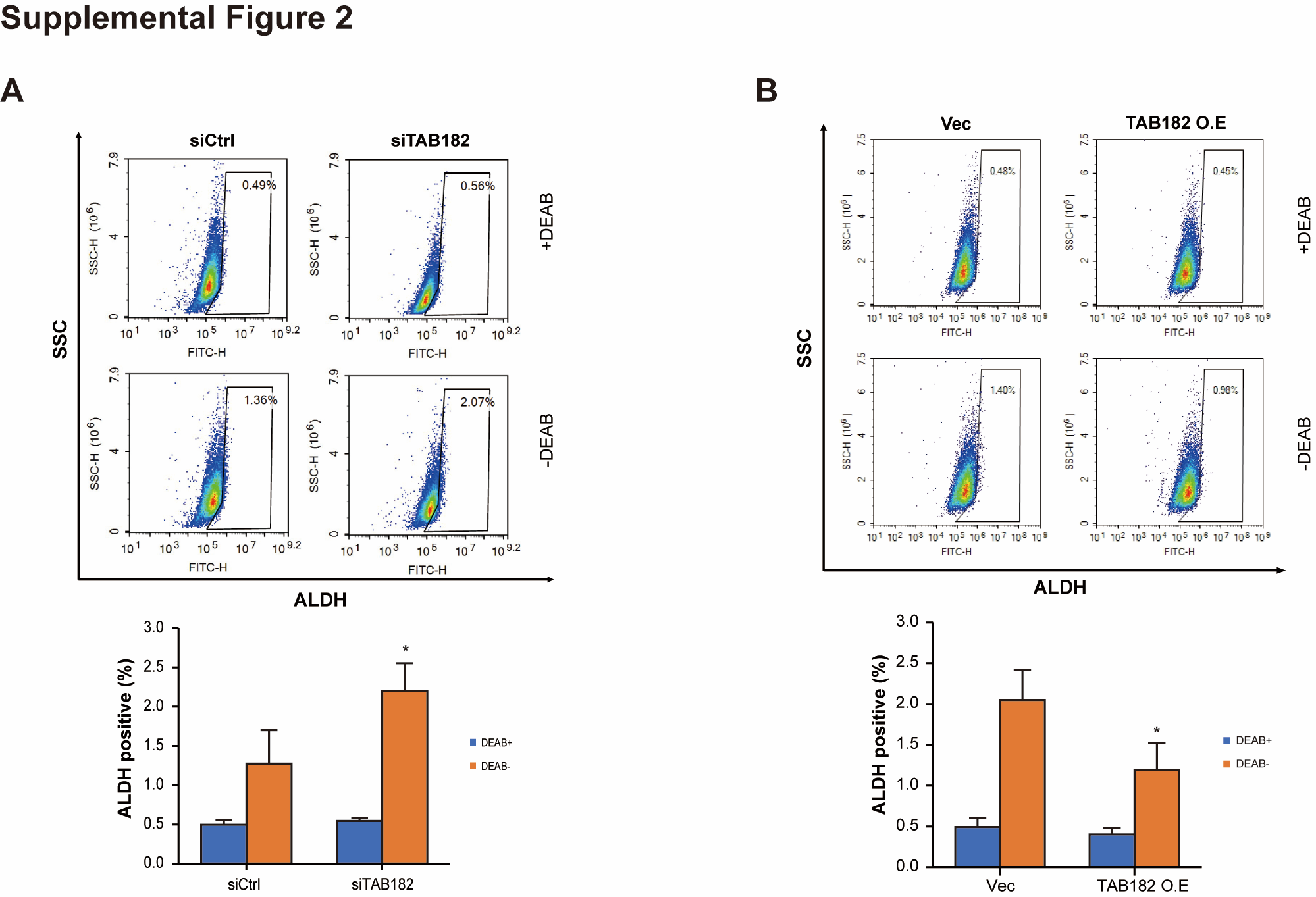


**Fig. S2. TAB182 deletion increases the percentage of ALDH-positive cells.** ALDEFLUOR assay was performed to detect changes in percentages of the ALDH-positive population in BT549 cells upon TAB182 deletion **(A)** or overexpression **(B)**. DEAB was used as the negative control. SSC: side scatter. FITH: the fluorescence signal of ALDH. Percentages of ALDH positive cells were presented at the bottom of A and B. ^*^ *P* < 0.05, DEAB- siTAB182/TAB182 O.E vs. DEAB- siCtrl/Vec, by Student’s t test.


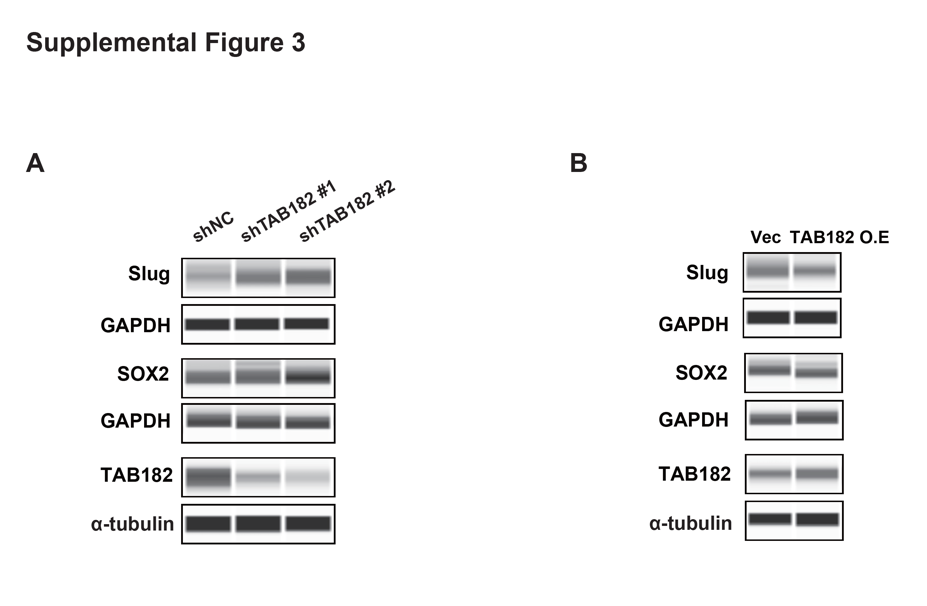


**Figure S3. Deleting TAB182 enhances the expression of cancer stemness-related protein markers.** Western blot analysis of protein levels of cancer stem cell markers, including SOX2 and Slug, after deleting **(A)** or overexpressing TAB182 **(B)** in MDA-MB-231 cells. TAB182 was used to validate the knockdown or overexpression effects, and GAPDH or 𝛼-tubulin was used as the loading control.
